# Supplementary material for: BEEtag: A Low-Cost, Image-Based Tracking System for the Study of Animal Behavior and Locomotion
Source: PLoS One. 2015 Sep 2;10(9):e0136487. doi: 10.1371/journal.pone.0136487 (PMC4558030; doi:10.1371/journal.pone.0136487)
Supplement: S1 Code Supplement — Functions and dependencies associated with the BEEtag tracking software for Matlab. (ZIP) [file pone.0136487.s001.zip › BEEtag-master/src/0-99keyed.pdf]

1 ->	4 ->	9 ->	11 ->	14 ->	15 ->	18 ->	32 ->	33 ->	36 ->
37 ->	42 ->	43 ->	46 ->	47 ->	66 ->	67 ->	70 ->	71 ->	72 ->
73 ->	76 ->	77 ->	98 ->	101 ->	103 ->	104 ->	106 ->	108 ->	109 ->
119 ->	128 ->	129 ->	133 ->	135 ->	136 ->	138 ->	142 ->	143 ->	160 ->
161 ->	164 ->	166 ->	169 ->	171 ->	174 ->	175 ->	194 ->	195 ->	197 ->
199 ->	200 ->	202 ->	204 ->	205 ->	226 ->	227 ->	228 ->	230 ->	233 ->
235 ->	236 ->	237 ->	258 ->	259 ->	260 ->	262 ->	265 ->	267 ->	268 ->
269 ->	290 ->	291 ->	293 ->	295 ->	296 ->	298 ->	300 ->	301 ->	320 ->
321 ->	324 ->	326 ->	329 ->	331 ->	334 ->	335 ->	352 ->	353 ->	357 ->
359 ->	360 ->	362 ->	366 ->	367 ->	386 ->	389 ->	391 ->	392 ->	394 ->
