# Supplementary material for: BEEtag: A Low-Cost, Image-Based Tracking System for the Study of Animal Behavior and Locomotion
Source: PLoS One. 2015 Sep 2;10(9):e0136487. doi: 10.1371/journal.pone.0136487 (PMC4558030; doi:10.1371/journal.pone.0136487)
Supplement: S1 Code Supplement — Functions and dependencies associated with the BEEtag tracking software for Matlab. (ZIP) [file pone.0136487.s001.zip › BEEtag-master/src/100-199keyed.pdf]

397 ->	407 ->	418 ->	419 ->	422 ->	423 ->	424 ->	425 ->	428 ->	429 ->
448 ->	449 ->	452 ->	453 ->	458 ->	459 ->	462 ->	463 ->	480 ->	481 ->
484 ->	486 ->	489 ->	491 ->	494 ->	495 ->	528 ->	529 ->	532 ->	533 ->
538 ->	539 ->	542 ->	543 ->	560 ->	561 ->	564 ->	565 ->	570 ->	571 ->
575 ->	594 ->	595 ->	598 ->	599 ->	600 ->	601 ->	604 ->	605 ->	626 ->
627 ->	630 ->	631 ->	632 ->	633 ->	636 ->	637 ->	643 ->	646 ->	656 ->
657 ->	661 ->	664 ->	666 ->	671 ->	688 ->	689 ->	692 ->	693 ->	698 ->
699 ->	702 ->	703 ->	722 ->	723 ->	726 ->	727 ->	728 ->	729 ->	732 ->
733 ->	754 ->	755 ->	756 ->	758 ->	761 ->	763 ->	764 ->	765 ->	787 ->
788 ->	790 ->	793 ->	795 ->	796 ->	797 ->	818 ->	819 ->	822 ->	823 ->
