# Supplementary material for: BEEtag: A Low-Cost, Image-Based Tracking System for the Study of Animal Behavior and Locomotion
Source: PLoS One. 2015 Sep 2;10(9):e0136487. doi: 10.1371/journal.pone.0136487 (PMC4558030; doi:10.1371/journal.pone.0136487)
Supplement: S1 Code Supplement — Functions and dependencies associated with the BEEtag tracking software for Matlab. (ZIP) [file pone.0136487.s001.zip › BEEtag-master/src/200-299keyed.pdf]

824 ->	825 ->	828 ->	829 ->	848 ->	849 ->	852 ->	853 ->	858 ->	859 ->
863 ->	867 ->	880 ->	881 ->	885 ->	888 ->	890 ->	894 ->	895 ->	914 ->
915 ->	918 ->	919 ->	921 ->	924 ->	925 ->	946 ->	947 ->	950 ->	951 ->
952 ->	953 ->	956 ->	957 ->	976 ->	977 ->	980 ->	981 ->	986 ->	987 ->
990 ->	991 ->	1008 ->	1009 ->	1012 ->	1013 ->	1018 ->	1019 ->	1022 ->	1023 ->
1024 ->	1025 ->	1028 ->	1029 ->	1034 ->	1035 ->	1038 ->	1039 ->	1056 ->	1057 ->
1060 ->	1061 ->	1066 ->	1067 ->	1070 ->	1071 ->	1090 ->	1091 ->	1094 ->	1095 ->
1096 ->	1097 ->	1100 ->	1101 ->	1122 ->	1123 ->	1126 ->	1127 ->	1128 ->	1129 ->
1132 ->	1133 ->	1152 ->	1153 ->	1156 ->	1157 ->	1162 ->	1163 ->	1166 ->	1167 ->
1184 ->	1185 ->	1188 ->	1189 ->	1194 ->	1195 ->	1198 ->	1199 ->	1218 ->	1219 ->
