# Supplementary material for: BEEtag: A Low-Cost, Image-Based Tracking System for the Study of Animal Behavior and Locomotion
Source: PLoS One. 2015 Sep 2;10(9):e0136487. doi: 10.1371/journal.pone.0136487 (PMC4558030; doi:10.1371/journal.pone.0136487)
Supplement: S1 Code Supplement — Functions and dependencies associated with the BEEtag tracking software for Matlab. (ZIP) [file pone.0136487.s001.zip › BEEtag-master/src/300-399keyed.pdf]

1222 ->	1223 ->	1224 ->	1225 ->	1228 ->	1229 ->	1250 ->	1251 ->	1254 ->	1255 ->
1256 ->	1257 ->	1260 ->	1261 ->	1282 ->	1283 ->	1286 ->	1287 ->	1288 ->	1289 ->
1292 ->	1293 ->	1314 ->	1315 ->	1318 ->	1319 ->	1320 ->	1321 ->	1324 ->	1325 ->
1344 ->	1345 ->	1348 ->	1349 ->	1354 ->	1355 ->	1358 ->	1359 ->	1376 ->	1377 ->
1380 ->	1381 ->	1386 ->	1387 ->	1390 ->	1391 ->	1410 ->	1411 ->	1414 ->	1415 ->
1416 ->	1417 ->	1420 ->	1421 ->	1442 ->	1443 ->	1446 ->	1447 ->	1448 ->	1449 ->
1452 ->	1453 ->	1472 ->	1473 ->	1476 ->	1477 ->	1482 ->	1483 ->	1486 ->	1487 ->
1504 ->	1505 ->	1508 ->	1509 ->	1514 ->	1515 ->	1518 ->	1519 ->	1552 ->	1553 ->
1556 ->	1557 ->	1562 ->	1563 ->	1566 ->	1567 ->	1584 ->	1585 ->	1588 ->	1589 ->
1594 ->	1595 ->	1598 ->	1599 ->	1618 ->	1619 ->	1622 ->	1623 ->	1624 ->	1625 ->
