# Supplementary material for: BEEtag: A Low-Cost, Image-Based Tracking System for the Study of Animal Behavior and Locomotion
Source: PLoS One. 2015 Sep 2;10(9):e0136487. doi: 10.1371/journal.pone.0136487 (PMC4558030; doi:10.1371/journal.pone.0136487)
Supplement: S1 Code Supplement — Functions and dependencies associated with the BEEtag tracking software for Matlab. (ZIP) [file pone.0136487.s001.zip › BEEtag-master/src/400-499keyed.pdf]

1628 ->	1629 ->	1650 ->	1651 ->	1654 ->	1655 ->	1656 ->	1657 ->	1660 ->	1661 ->
1680 ->	1681 ->	1684 ->	1685 ->	1690 ->	1691 ->	1694 ->	1695 ->	1712 ->	1713 ->
1716 ->	1717 ->	1722 ->	1723 ->	1726 ->	1727 ->	1746 ->	1747 ->	1750 ->	1751 ->
1752 ->	1753 ->	1756 ->	1757 ->	1778 ->	1779 ->	1782 ->	1783 ->	1784 ->	1785 ->
1788 ->	1789 ->	1810 ->	1811 ->	1814 ->	1815 ->	1816 ->	1817 ->	1820 ->	1821 ->
1842 ->	1843 ->	1846 ->	1847 ->	1848 ->	1849 ->	1852 ->	1853 ->	1872 ->	1873 ->
1876 ->	1877 ->	1882 ->	1883 ->	1886 ->	1887 ->	1904 ->	1905 ->	1908 ->	1909 ->
1914 ->	1915 ->	1918 ->	1919 ->	1938 ->	1939 ->	1942 ->	1943 ->	1944 ->	1945 ->
1948 ->	1949 ->	1970 ->	1971 ->	1974 ->	1975 ->	1976 ->	1977 ->	1980 ->	1981 ->
2000 ->	2001 ->	2004 ->	2005 ->	2010 ->	2011 ->	2014 ->	2015 ->	2032 ->	2033 ->
