# Supplementary material for: BEEtag: A Low-Cost, Image-Based Tracking System for the Study of Animal Behavior and Locomotion
Source: PLoS One. 2015 Sep 2;10(9):e0136487. doi: 10.1371/journal.pone.0136487 (PMC4558030; doi:10.1371/journal.pone.0136487)
Supplement: S1 Code Supplement — Functions and dependencies associated with the BEEtag tracking software for Matlab. (ZIP) [file pone.0136487.s001.zip › BEEtag-master/src/500-599keyed.pdf]

2036 ->	2037 ->	2042 ->	2043 ->	2046 ->	2047 ->	2051 ->	2054 ->	2055 ->	2056 ->
2057 ->	2060 ->	2061 ->	2070 ->	2082 ->	2083 ->	2086 ->	2087 ->	2088 ->	2089 ->
2092 ->	2093 ->	2112 ->	2113 ->	2116 ->	2117 ->	2122 ->	2123 ->	2126 ->	2127 ->
2144 ->	2145 ->	2148 ->	2149 ->	2154 ->	2155 ->	2158 ->	2159 ->	2178 ->	2179 ->
2182 ->	2183 ->	2184 ->	2185 ->	2188 ->	2189 ->	2210 ->	2211 ->	2214 ->	2215 ->
2216 ->	2217 ->	2220 ->	2221 ->	2240 ->	2241 ->	2244 ->	2245 ->	2250 ->	2251 ->
2254 ->	2255 ->	2272 ->	2273 ->	2276 ->	2277 ->	2282 ->	2283 ->	2286 ->	2287 ->
2304 ->	2305 ->	2308 ->	2309 ->	2314 ->	2315 ->	2318 ->	2319 ->	2336 ->	2337 ->
2340 ->	2346 ->	2347 ->	2350 ->	2351 ->	2353 ->	2370 ->	2371 ->	2374 ->	2375 ->
2376 ->	2377 ->	2380 ->	2381 ->	2402 ->	2403 ->	2406 ->	2407 ->	2408 ->	2409 ->
