# Supplementary figures and images for: BEEtag: A Low-Cost, Image-Based Tracking System for the Study of Animal Behavior and Locomotion
Source: PLoS One. 2015 Sep 2;10(9):e0136487. doi: 10.1371/journal.pone.0136487 (PMC4558030; doi:10.1371/journal.pone.0136487)

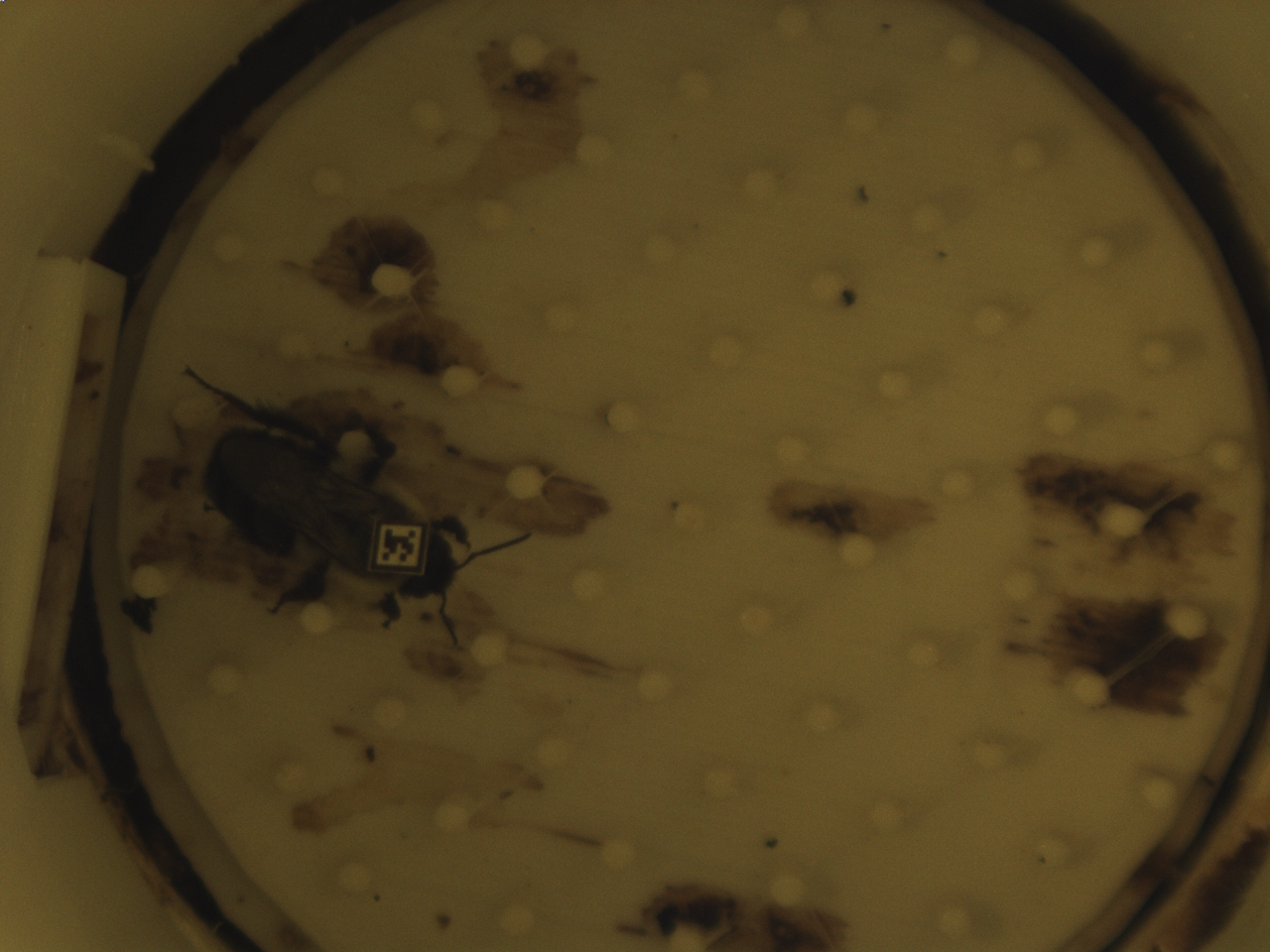

Supplement: S1 Code Supplement — Functions and dependencies associated with the BEEtag tracking software for Matlab. (ZIP) [file pone.0136487.s001.zip › BEEtag-master/scaleExample.png]

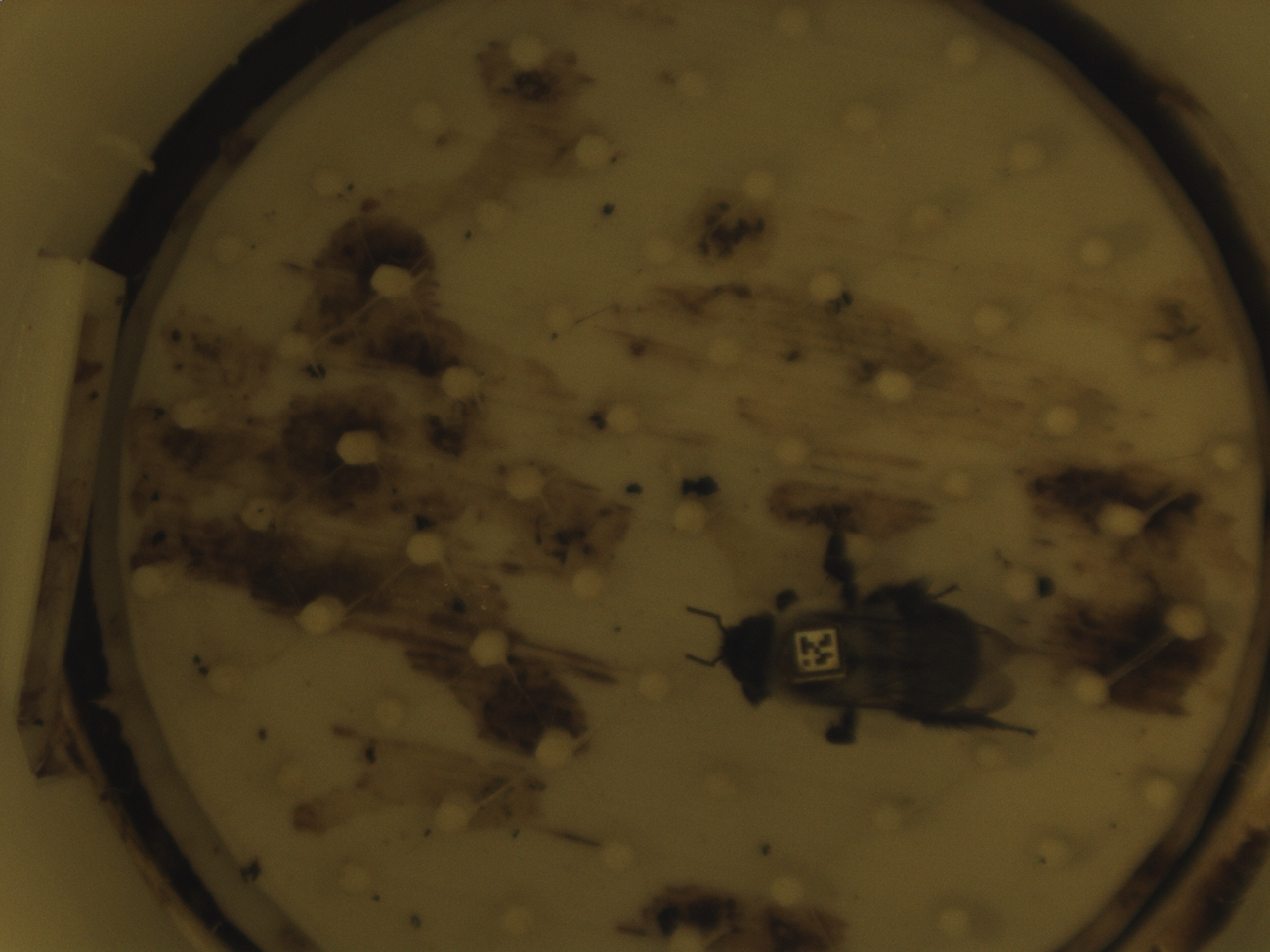

Supplement: S1 Code Supplement — Functions and dependencies associated with the BEEtag tracking software for Matlab. (ZIP) [file pone.0136487.s001.zip › BEEtag-master/scaleExample2.png]

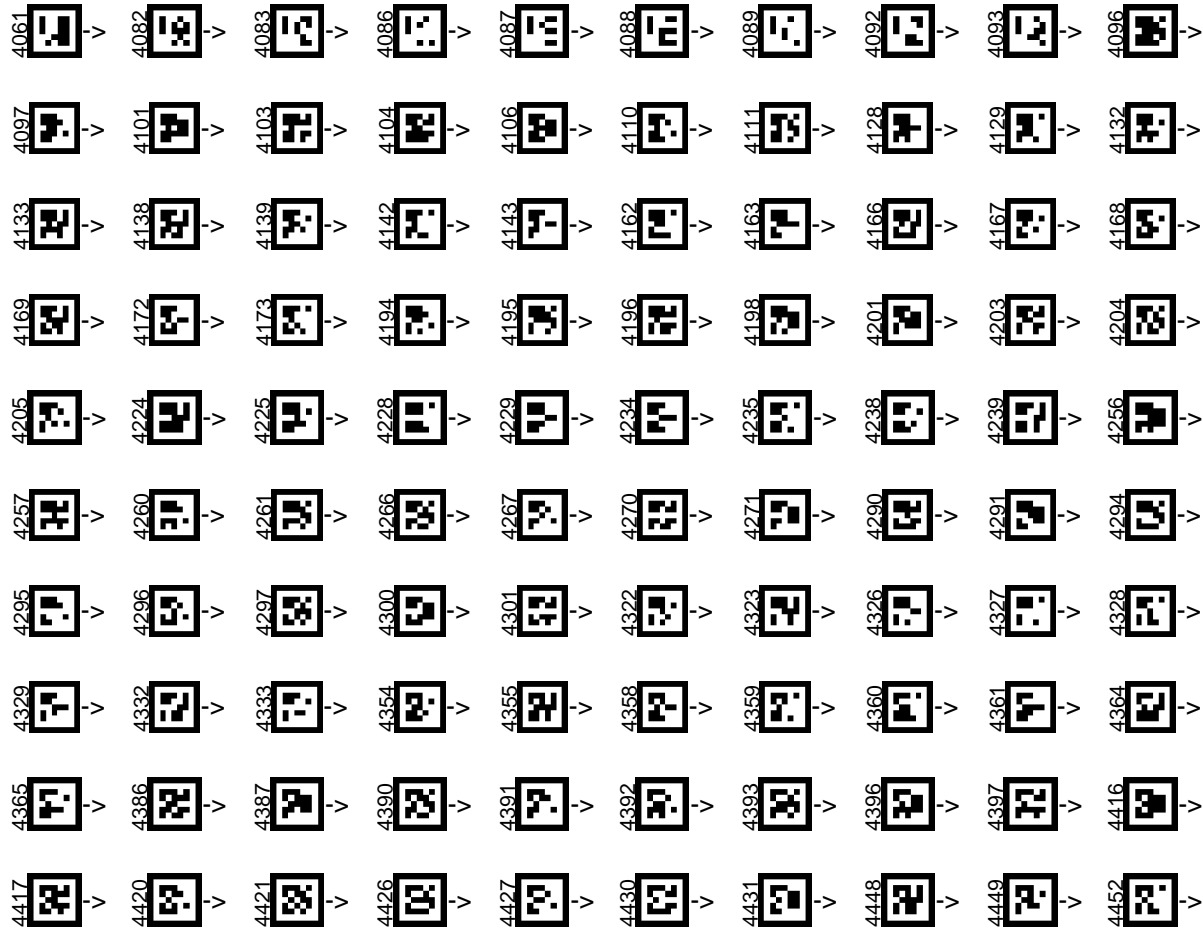

Supplement: S1 Code Supplement — Functions and dependencies associated with the BEEtag tracking software for Matlab. (ZIP) [file pone.0136487.s001.zip › BEEtag-master/src/1000-1099keyed.pdf]

|      |                                                                                     |      |                                                                                     |      |                                                                                     |      |                                                                                     |      |                                                                                     |      |                                                                                     |      |                                                                                      |      |                                                                                       |      |                                                                                       |      |                                                                                       |
|------|-------------------------------------------------------------------------------------|------|-------------------------------------------------------------------------------------|------|-------------------------------------------------------------------------------------|------|-------------------------------------------------------------------------------------|------|-------------------------------------------------------------------------------------|------|-------------------------------------------------------------------------------------|------|--------------------------------------------------------------------------------------|------|---------------------------------------------------------------------------------------|------|---------------------------------------------------------------------------------------|------|---------------------------------------------------------------------------------------|
| 4453 | 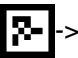   | 4458 | 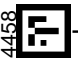   | 4459 | 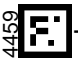   | 4462 | 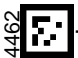   | 4463 | 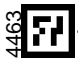   | 4482 | 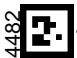   | 4483 | 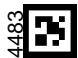   | 4484 | 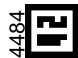   | 4486 | 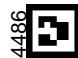   | 4489 | 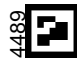   |
| 4491 | 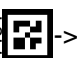   | 4492 | 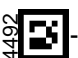   | 4493 | 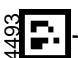   | 4514 | 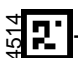   | 4515 | 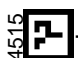   | 4518 | 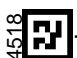   | 4519 | 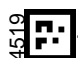   | 4520 | 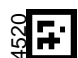   | 4521 | 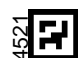   | 4524 | 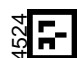   |
| 4525 | 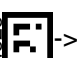   | 4544 | 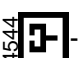   | 4545 | 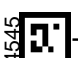   | 4548 | 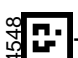   | 4549 | 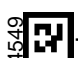   | 4554 | 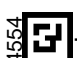   | 4555 | 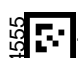   | 4558 | 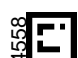   | 4559 | 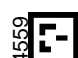   | 4576 | 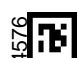   |
| 4577 | 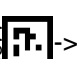   | 4581 | 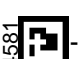   | 4583 | 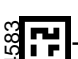   | 4584 | 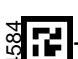   | 4586 | 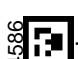   | 4590 | 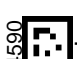   | 4591 | 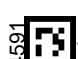   | 4624 | 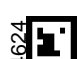   | 4625 | 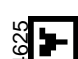   | 4628 | 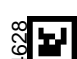   |
| 4629 | 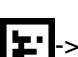   | 4634 | 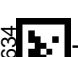   | 4635 | 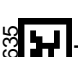   | 4638 | 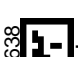   | 4639 | 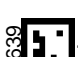   | 4656 | 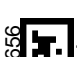   | 4657 | 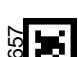   | 4660 | 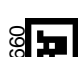   | 4661 | 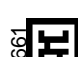   | 4666 | 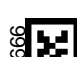   |
| 4667 | 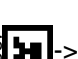 | 4670 | 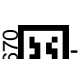 | 4671 | 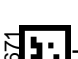 | 4690 | 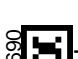 | 4691 | 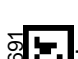 | 4694 | 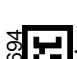 | 4695 | 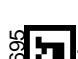 | 4696 | 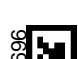 | 4697 | 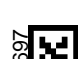 | 4700 | 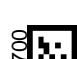 |
| 4701 | 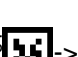 | 4722 | 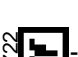 | 4723 | 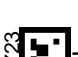 | 4726 | 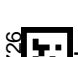 | 4727 | 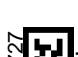 | 4728 | 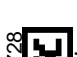 | 4729 | 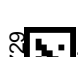 | 4732 | 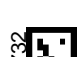 | 4733 | 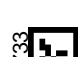 | 4752 | 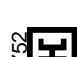 |
| 4753 | 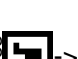 | 4756 | 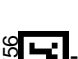 | 4757 | 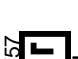 | 4762 | 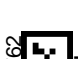 | 4763 | 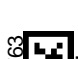 | 4766 | 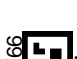 | 4767 | 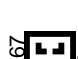 | 4784 | 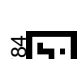 | 4785 | 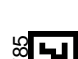 | 4788 | 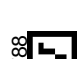 |
| 4789 | 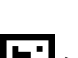 | 4794 | 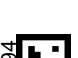 | 4795 | 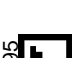 | 4798 | 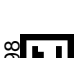 | 4799 | 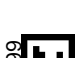 | 4818 | 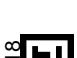 | 4819 | 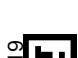 | 4822 | 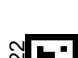 | 4823 | 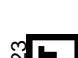 | 4824 | 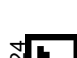 |
| 4825 | 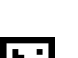 | 4828 | 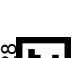 | 4829 | 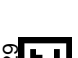 | 4850 | 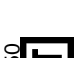 | 4851 | 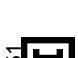 | 4854 | 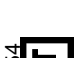 | 4855 | 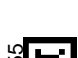 | 4856 | 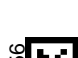 | 4857 | 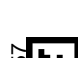 | 4860 | 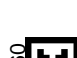 |

Supplement: S1 Code Supplement — Functions and dependencies associated with the BEEtag tracking software for Matlab. (ZIP) [file pone.0136487.s001.zip › BEEtag-master/src/1100-1199keyed.pdf]

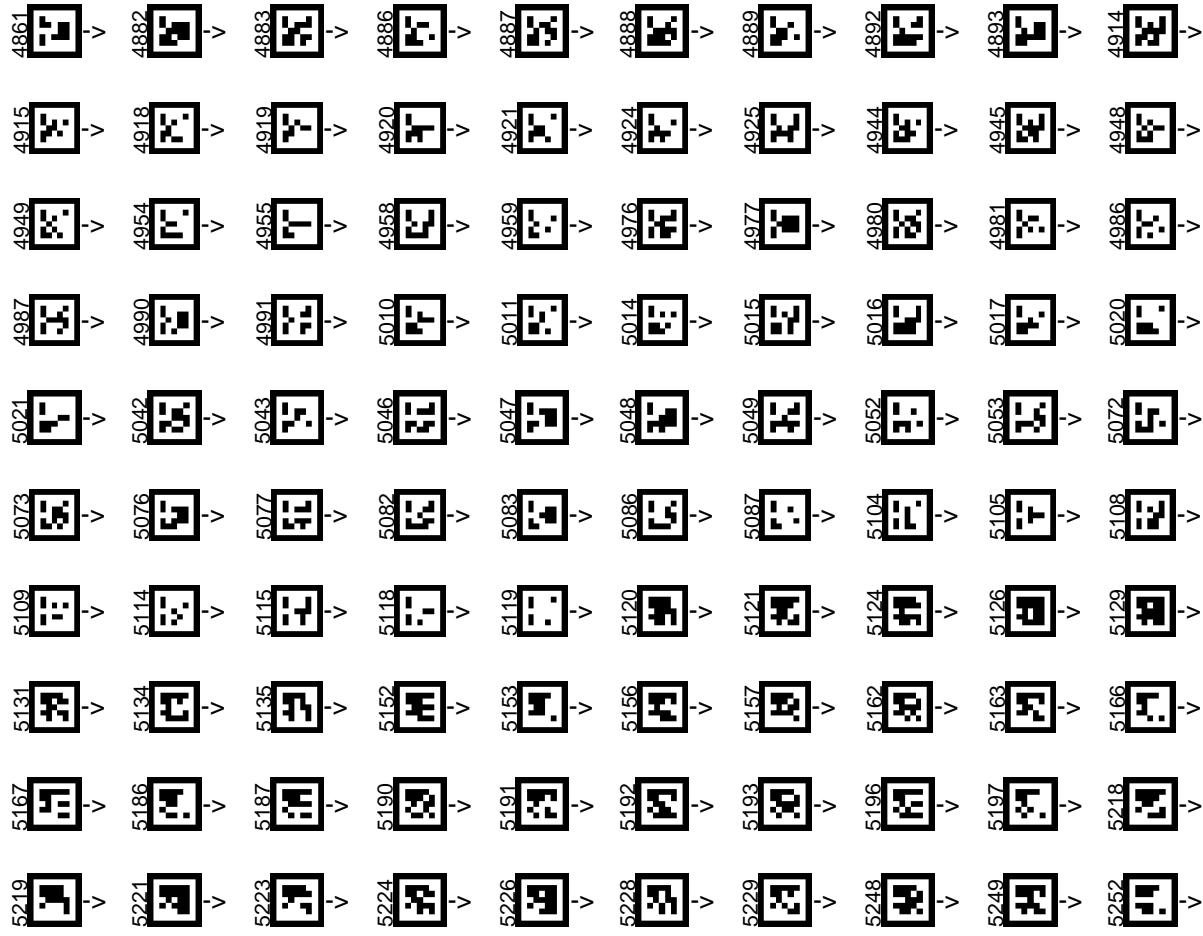

Supplement: S1 Code Supplement — Functions and dependencies associated with the BEEtag tracking software for Matlab. (ZIP) [file pone.0136487.s001.zip › BEEtag-master/src/1200-1299keyed.pdf]

|      |                                                                                     |      |                                                                                     |      |                                                                                     |      |                                                                                     |      |                                                                                     |      |                                                                                     |      |                                                                                     |      |                                                                                       |      |                                                                                       |      |                                                                                       |
|------|-------------------------------------------------------------------------------------|------|-------------------------------------------------------------------------------------|------|-------------------------------------------------------------------------------------|------|-------------------------------------------------------------------------------------|------|-------------------------------------------------------------------------------------|------|-------------------------------------------------------------------------------------|------|-------------------------------------------------------------------------------------|------|---------------------------------------------------------------------------------------|------|---------------------------------------------------------------------------------------|------|---------------------------------------------------------------------------------------|
| 5253 | 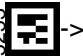   | 5258 | 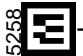   | 5259 | 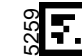   | 5262 | 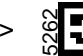   | 5263 | 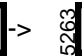   | 5281 | 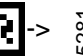   | 5283 | 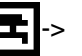   | 5284 | 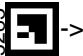   | 5285 | 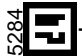   | 5290 | 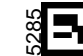   |
| 5291 | 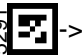   | 5292 | 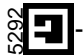   | 5294 | 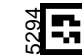   | 5312 | 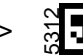   | 5314 | 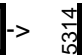   | 5318 | 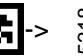   | 5319 | 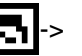   | 5320 | 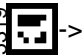   | 5321 | 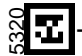   | 5325 | 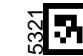   |
| 5327 | 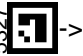   | 5346 | 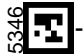   | 5347 | 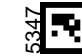   | 5350 | 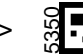   | 5351 | 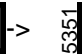   | 5352 | 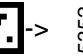   | 5353 | 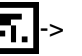   | 5356 | 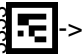   | 5357 | 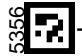   | 5378 | 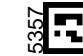   |
| 5379 | 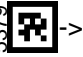   | 5382 | 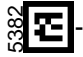   | 5383 | 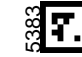   | 5384 | 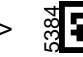   | 5385 | 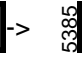   | 5388 | 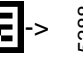   | 5389 | 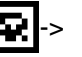   | 5408 | 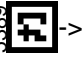   | 5410 | 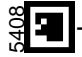   | 5414 | 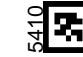   |
| 5415 | 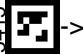  | 5416 | 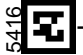  | 5417 | 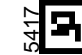  | 5421 | 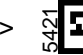  | 5423 | 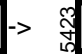  | 5441 | 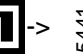  | 5443 | 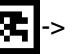  | 5444 | 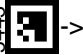  | 5445 | 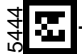  | 5450 | 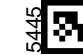  |
| 5451 | 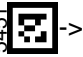 | 5454 | 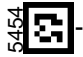 | 5460 | 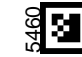 | 5472 | 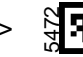 | 5473 | 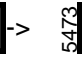 | 5476 | 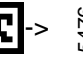 | 5477 | 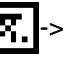 | 5482 | 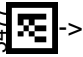 | 5483 | 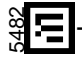 | 5486 | 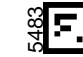 |
| 5487 | 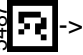 | 5506 | 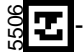 | 5507 | 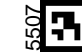 | 5509 | 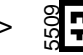 | 5511 | 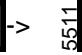 | 5512 | 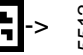 | 5514 | 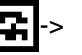 | 5516 | 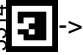 | 5517 | 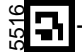 | 5538 | 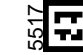 |
| 5539 | 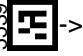 | 5542 | 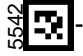 | 5543 | 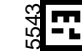 | 5544 | 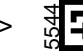 | 5545 | 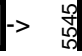 | 5548 | 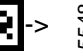 | 5549 | 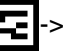 | 5568 | 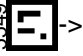 | 5569 | 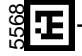 | 5572 | 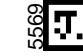 |
| 5573 | 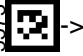 | 5578 | 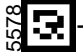 | 5579 | 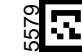 | 5582 | 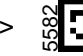 | 5583 | 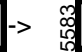 | 5600 | 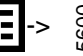 | 5601 | 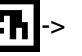 | 5604 | 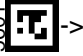 | 5606 | 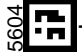 | 5609 | 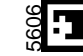 |
| 5611 | 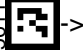 | 5614 | 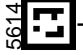 | 5615 | 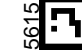 | 5648 | 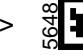 | 5649 | 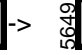 | 5652 | 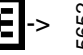 | 5653 | 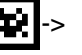 | 5658 | 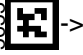 | 5659 | 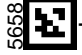 | 5662 | 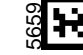 |

Supplement: S1 Code Supplement — Functions and dependencies associated with the BEEtag tracking software for Matlab. (ZIP) [file pone.0136487.s001.zip › BEEtag-master/src/1300-1399keyed.pdf]

|      |                                                                                     |      |                                                                                     |      |                                                                                     |      |                                                                                     |      |                                                                                     |      |                                                                                     |      |                                                                                     |      |                                                                                       |      |                                                                                       |      |                                                                                       |
|------|-------------------------------------------------------------------------------------|------|-------------------------------------------------------------------------------------|------|-------------------------------------------------------------------------------------|------|-------------------------------------------------------------------------------------|------|-------------------------------------------------------------------------------------|------|-------------------------------------------------------------------------------------|------|-------------------------------------------------------------------------------------|------|---------------------------------------------------------------------------------------|------|---------------------------------------------------------------------------------------|------|---------------------------------------------------------------------------------------|
| 5663 | 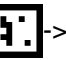   | 5680 | 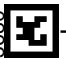   | 5681 | 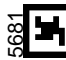   | 5684 | 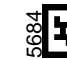   | 5685 | 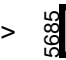   | 5690 | 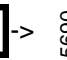   | 5691 | 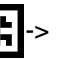   | 5694 | 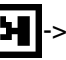   | 5695 | 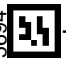   | 5708 | 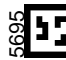   |
| 5714 | 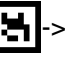   | 5715 | 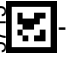   | 5718 | 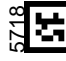   | 5719 | 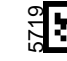   | 5721 | 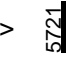   | 5724 | 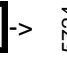   | 5725 | 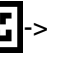   | 5746 | 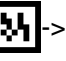   | 5747 | 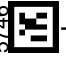   | 5750 | 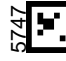   |
| 5751 | 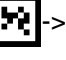   | 5752 | 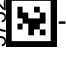   | 5753 | 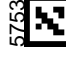   | 5756 | 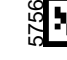   | 5757 | 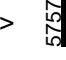   | 5781 | 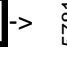   | 5784 | 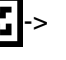   | 5789 | 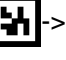   | 5808 | 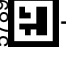   | 5809 | 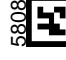   |
| 5812 | 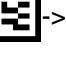   | 5813 | 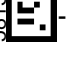   | 5818 | 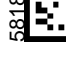   | 5819 | 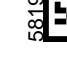   | 5822 | 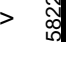   | 5823 | 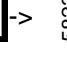   | 5842 | 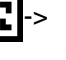   | 5843 | 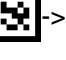   | 5846 | 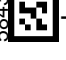   | 5847 | 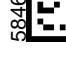   |
| 5848 | 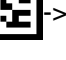  | 5849 | 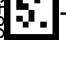  | 5852 | 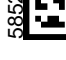  | 5853 | 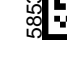  | 5872 | 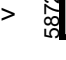  | 5873 | 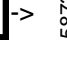  | 5876 | 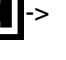  | 5878 | 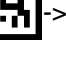  | 5881 | 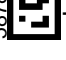  | 5883 | 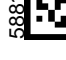  |
| 5886 | 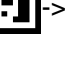 | 5887 | 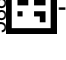 | 5904 | 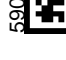 | 5905 | 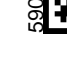 | 5908 | 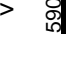 | 5910 | 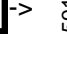 | 5913 | 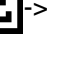 | 5915 | 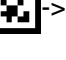 | 5918 | 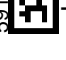 | 5919 | 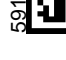 |
| 5938 | 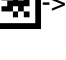 | 5939 | 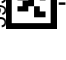 | 5942 | 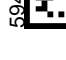 | 5943 | 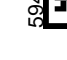 | 5944 | 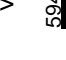 | 5945 | 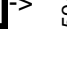 | 5948 | 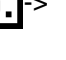 | 5949 | 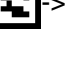 | 5968 | 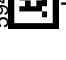 | 5969 | 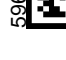 |
| 5972 | 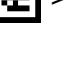 | 5973 | 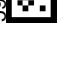 | 5978 | 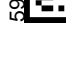 | 5979 | 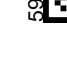 | 5982 | 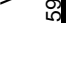 | 5983 | 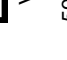 | 6002 | 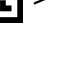 | 6003 | 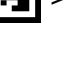 | 6005 | 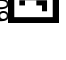 | 6007 | 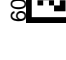 |
| 6008 | 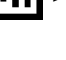 | 6010 | 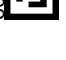 | 6012 | 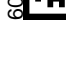 | 6013 | 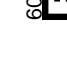 | 6034 | 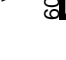 | 6035 | 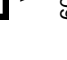 | 6038 | 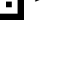 | 6039 | 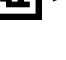 | 6040 | 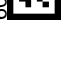 | 6041 | 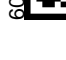 |
| 6044 | 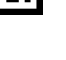 | 6045 | 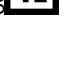 | 6066 | 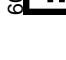 | 6067 | 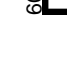 | 6070 | 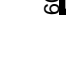 | 6071 | 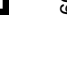 | 6072 | 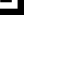 | 6073 | 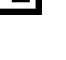 | 6076 | 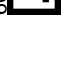 | 6077 | 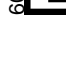 |

Supplement: S1 Code Supplement — Functions and dependencies associated with the BEEtag tracking software for Matlab. (ZIP) [file pone.0136487.s001.zip › BEEtag-master/src/1400-1499keyed.pdf]

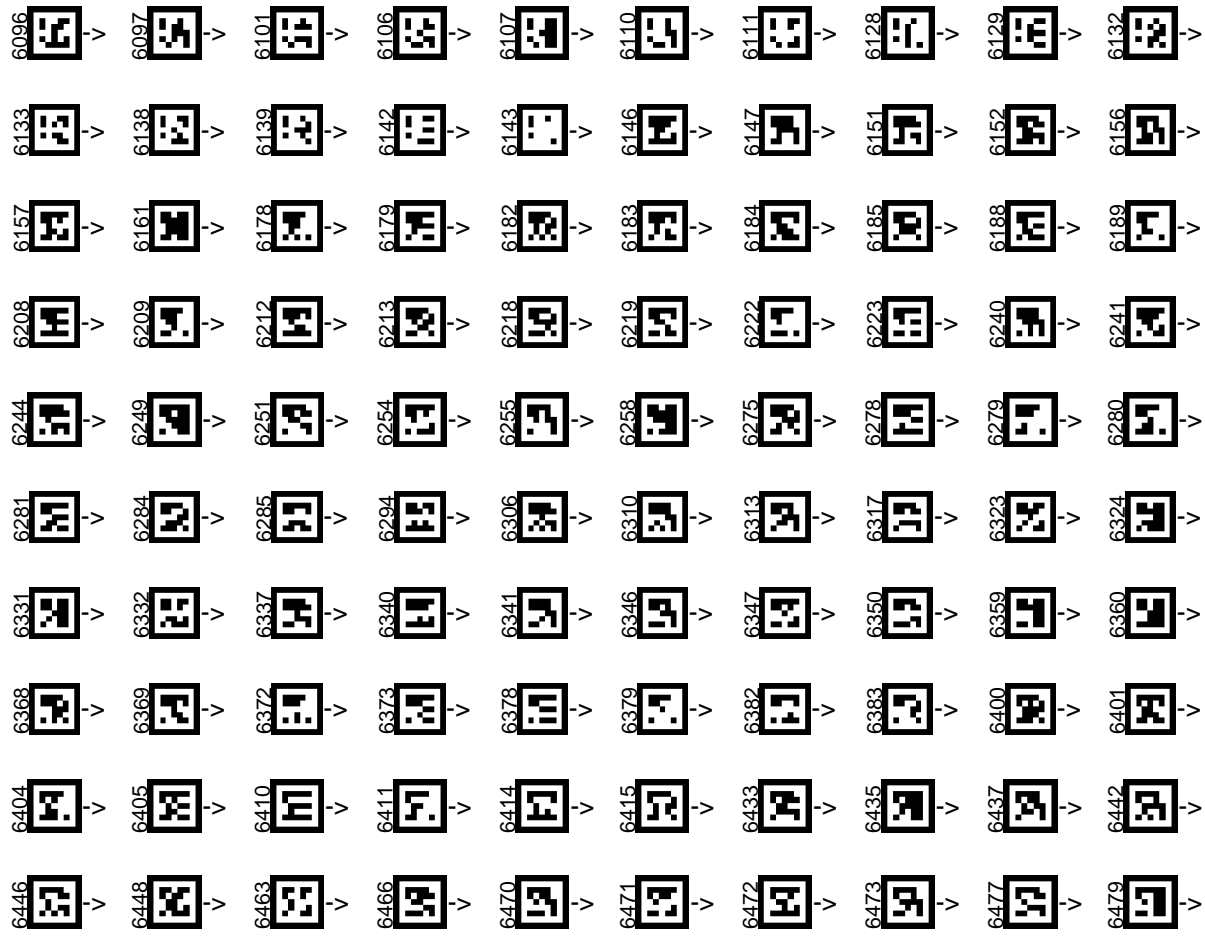

Supplement: S1 Code Supplement — Functions and dependencies associated with the BEEtag tracking software for Matlab. (ZIP) [file pone.0136487.s001.zip › BEEtag-master/src/1500-1599keyed.pdf]

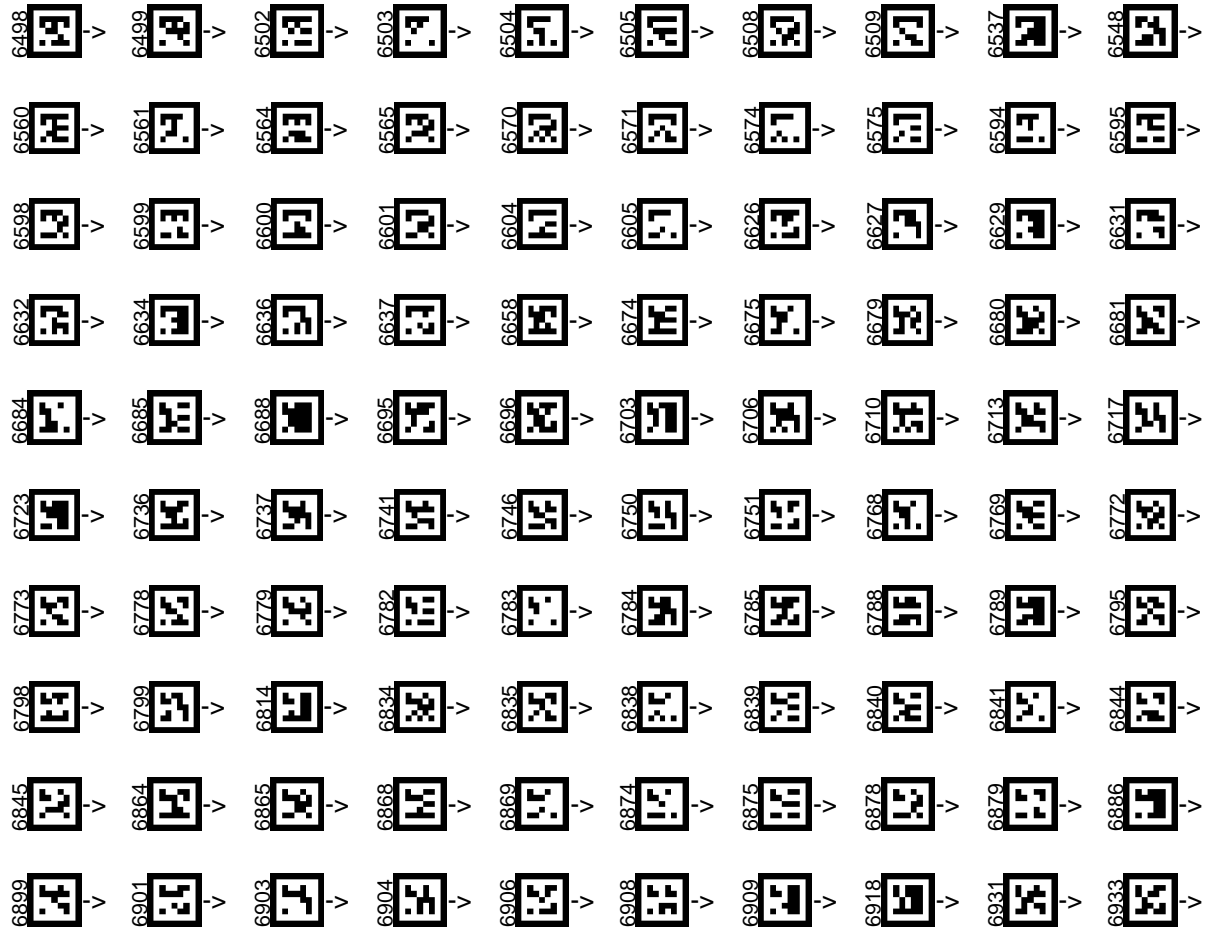

Supplement: S1 Code Supplement — Functions and dependencies associated with the BEEtag tracking software for Matlab. (ZIP) [file pone.0136487.s001.zip › BEEtag-master/src/1600-1699keyed.pdf]

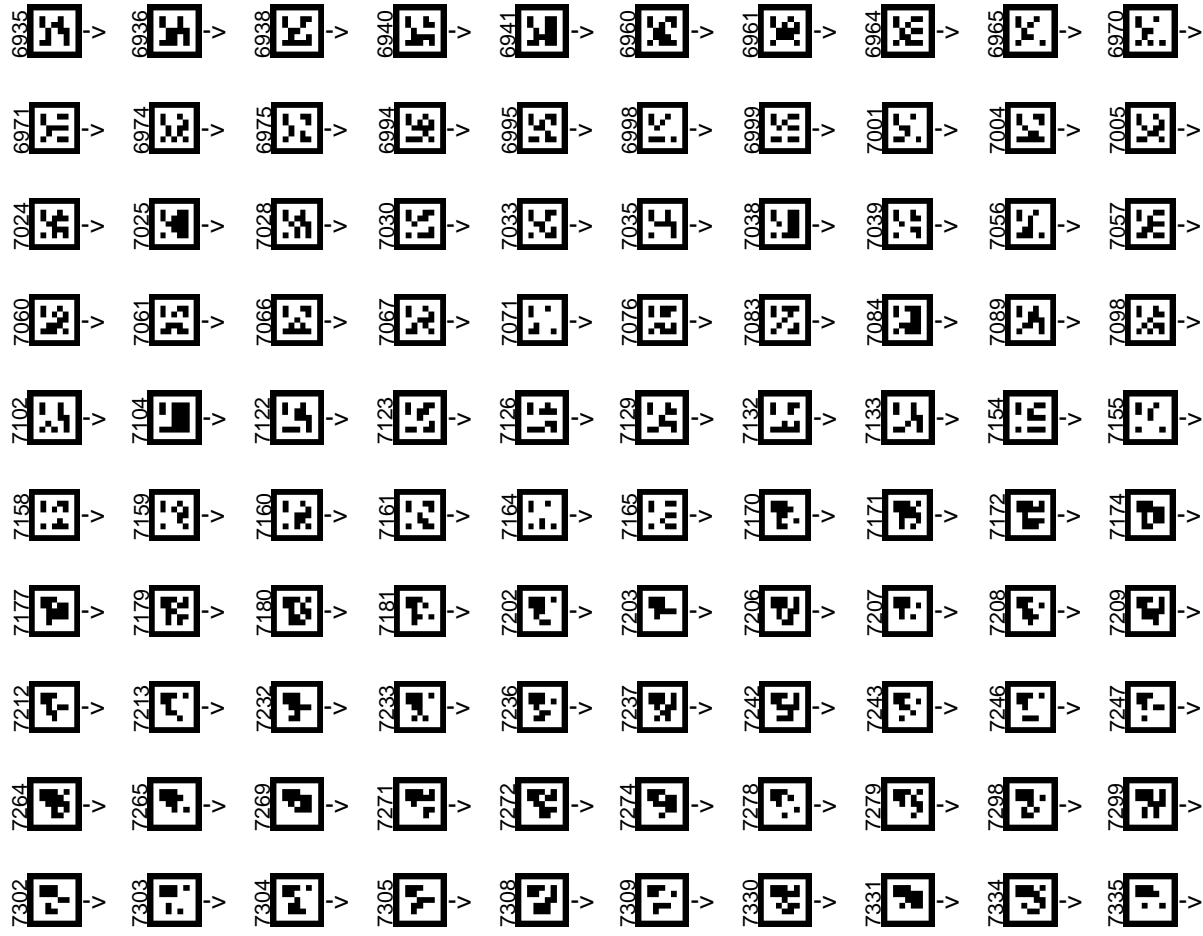

Supplement: S1 Code Supplement — Functions and dependencies associated with the BEEtag tracking software for Matlab. (ZIP) [file pone.0136487.s001.zip › BEEtag-master/src/1700-1799keyed.pdf]

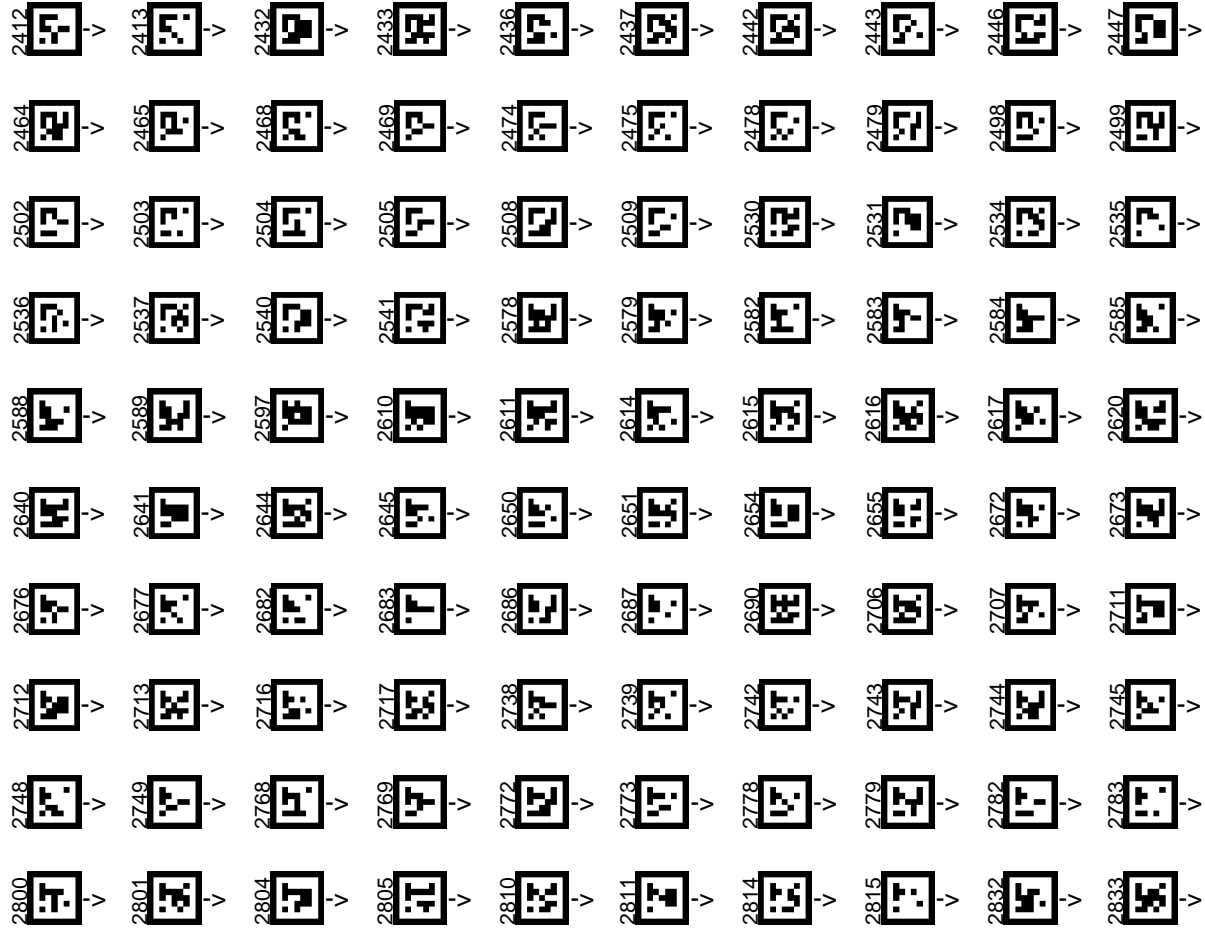

Supplement: S1 Code Supplement — Functions and dependencies associated with the BEEtag tracking software for Matlab. (ZIP) [file pone.0136487.s001.zip › BEEtag-master/src/600-699keyed.pdf]

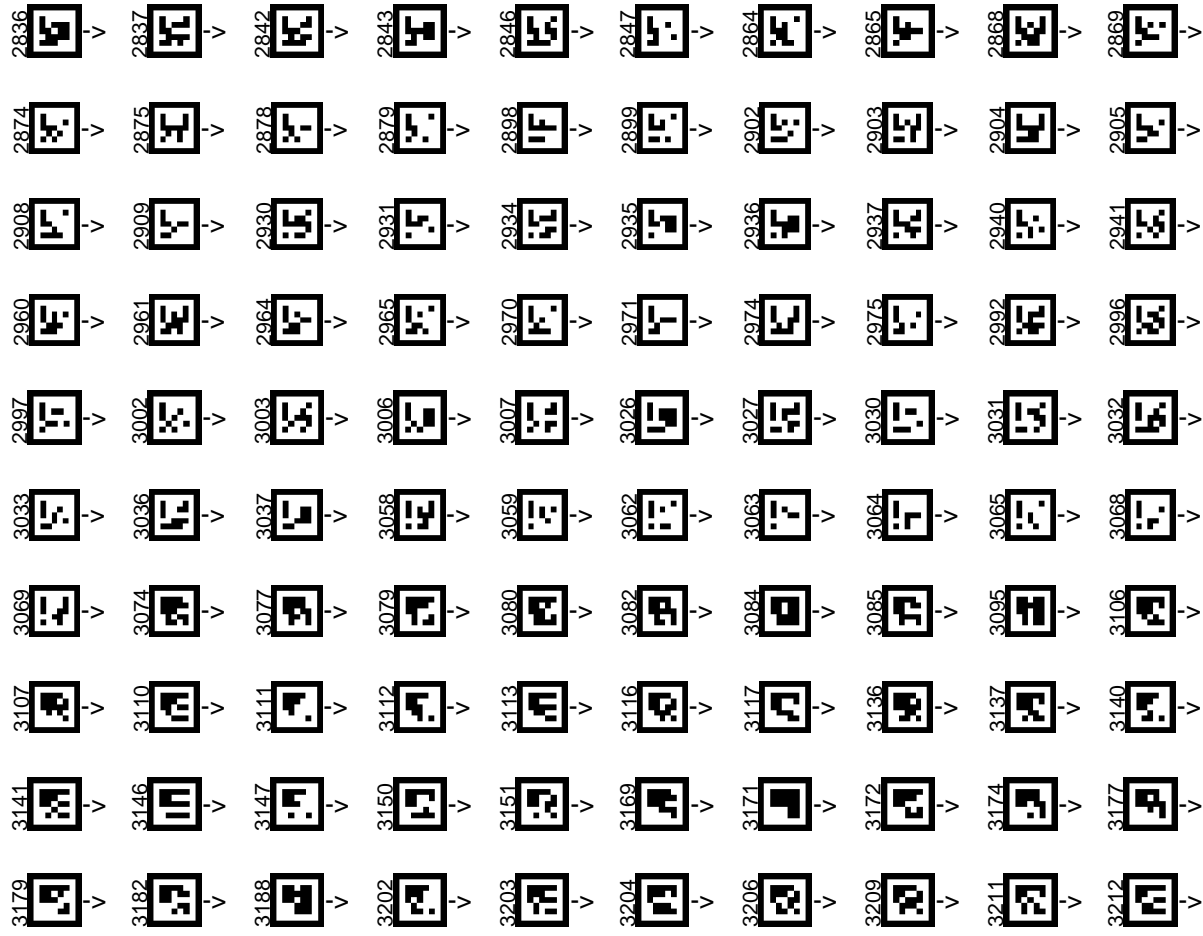

Supplement: S1 Code Supplement — Functions and dependencies associated with the BEEtag tracking software for Matlab. (ZIP) [file pone.0136487.s001.zip › BEEtag-master/src/700-799keyed.pdf]

|      |                                                                                     |      |                                                                                     |      |                                                                                     |      |                                                                                     |      |                                                                                     |      |                                                                                     |      |                                                                                      |      |                                                                                       |      |                                                                                       |      |                                                                                       |
|------|-------------------------------------------------------------------------------------|------|-------------------------------------------------------------------------------------|------|-------------------------------------------------------------------------------------|------|-------------------------------------------------------------------------------------|------|-------------------------------------------------------------------------------------|------|-------------------------------------------------------------------------------------|------|--------------------------------------------------------------------------------------|------|---------------------------------------------------------------------------------------|------|---------------------------------------------------------------------------------------|------|---------------------------------------------------------------------------------------|
| 3213 | 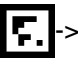   | 3234 | 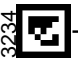   | 3235 | 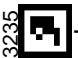   | 3237 | 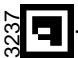   | 3239 | 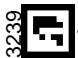   | 3240 | 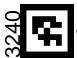   | 3242 | 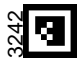   | 3244 | 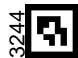   | 3245 | 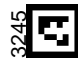   | 3264 | 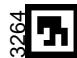   |
| 3265 | 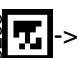   | 3268 | 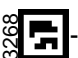   | 3270 | 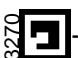   | 3273 | 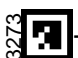   | 3275 | 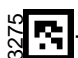   | 3278 | 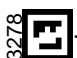   | 3279 | 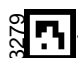   | 3296 | 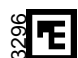   | 3297 | 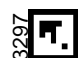   | 3301 | 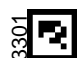   |
| 3303 | 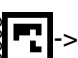   | 3304 | 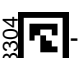   | 3306 | 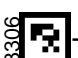   | 3310 | 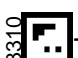   | 3311 | 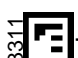   | 3328 | 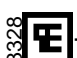   | 3329 | 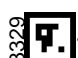   | 3333 | 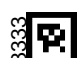   | 3335 | 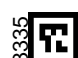   | 3336 | 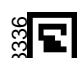   |
| 3338 | 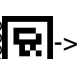   | 3342 | 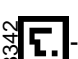   | 3343 | 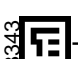   | 3360 | 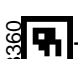   | 3361 | 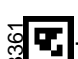   | 3364 | 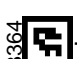   | 3366 | 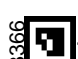   | 3371 | 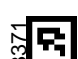   | 3374 | 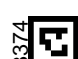   | 3375 | 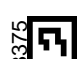   |
| 3377 | 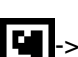   | 3394 | 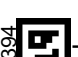   | 3395 | 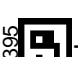   | 3397 | 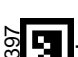   | 3399 | 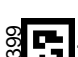   | 3400 | 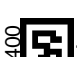   | 3402 | 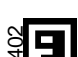   | 3404 | 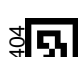   | 3405 | 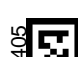   | 3426 | 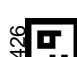   |
| 3427 | 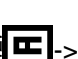 | 3428 | 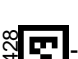 | 3430 | 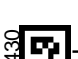 | 3433 | 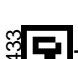 | 3435 | 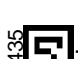 | 3436 | 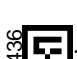 | 3437 | 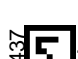 | 3456 | 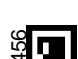 | 3457 | 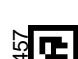 | 3460 | 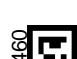 |
| 3462 | 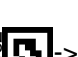 | 3465 | 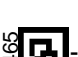 | 3467 | 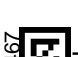 | 3470 | 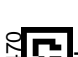 | 3471 | 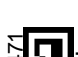 | 3488 | 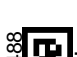 | 3489 | 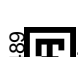 | 3492 | 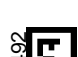 | 3493 | 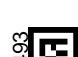 | 3498 | 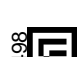 |
| 3499 | 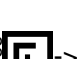 | 3502 | 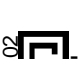 | 3503 | 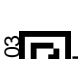 | 3522 | 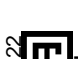 | 3523 | 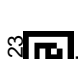 | 3526 | 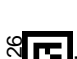 | 3527 | 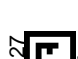 | 3528 | 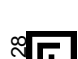 | 3529 | 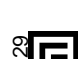 | 3532 | 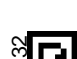 |
| 3533 | 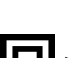 | 3554 | 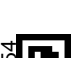 | 3557 | 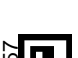 | 3559 | 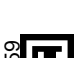 | 3560 | 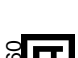 | 3562 | 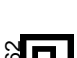 | 3564 | 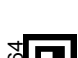 | 3565 | 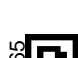 | 3575 | 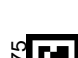 | 3602 | 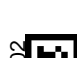 |
| 3603 | 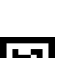 | 3606 | 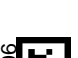 | 3607 | 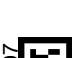 | 3608 | 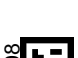 | 3609 | 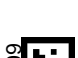 | 3612 | 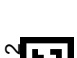 | 3613 | 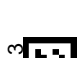 | 3635 | 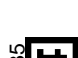 | 3638 | 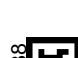 | 3639 | 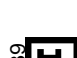 |

Supplement: S1 Code Supplement — Functions and dependencies associated with the BEEtag tracking software for Matlab. (ZIP) [file pone.0136487.s001.zip › BEEtag-master/src/800-899keyed.pdf]

|      |                                                                                     |   |      |                                                                                     |   |      |                                                                                     |   |      |                                                                                     |   |      |                                                                                     |   |      |                                                                                     |   |      |                                                                                      |   |      |                                                                                       |   |      |                                                                                       |   |      |                                                                                       |   |
|------|-------------------------------------------------------------------------------------|---|------|-------------------------------------------------------------------------------------|---|------|-------------------------------------------------------------------------------------|---|------|-------------------------------------------------------------------------------------|---|------|-------------------------------------------------------------------------------------|---|------|-------------------------------------------------------------------------------------|---|------|--------------------------------------------------------------------------------------|---|------|---------------------------------------------------------------------------------------|---|------|---------------------------------------------------------------------------------------|---|------|---------------------------------------------------------------------------------------|---|
| 3640 | 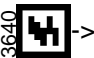   | ↘ | 3641 | 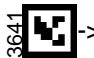   | ↘ | 3644 | 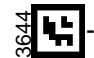   | ↘ | 3645 | 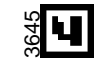   | ↘ | 3664 | 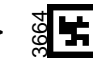   | ↘ | 3665 | 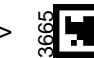   | ↘ | 3668 | 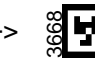   | ↘ | 3669 | 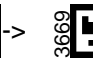   | ↘ | 3674 | 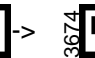   | ↘ | 3675 | 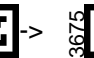   | ↘ |
| 3678 | 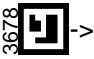   | ↘ | 3679 | 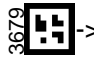   | ↘ | 3696 | 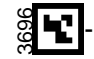   | ↘ | 3697 | 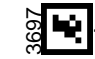   | ↘ | 3700 | 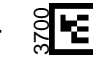   | ↘ | 3701 | 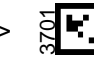   | ↘ | 3706 | 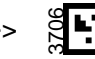   | ↘ | 3707 | 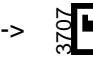   | ↘ | 3710 | 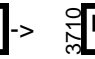   | ↘ | 3711 | 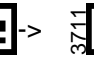   | ↘ |
| 3730 | 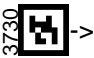   | ↘ | 3731 | 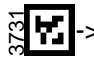   | ↘ | 3732 | 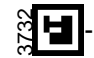   | ↘ | 3734 | 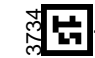   | ↘ | 3737 | 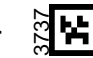   | ↘ | 3739 | 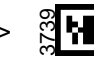   | ↘ | 3740 | 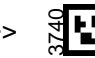   | ↘ | 3741 | 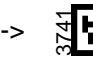   | ↘ | 3762 | 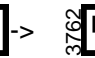   | ↘ | 3763 | 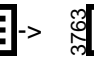   | ↘ |
| 3766 | 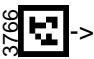   | ↘ | 3767 | 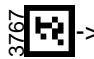   | ↘ | 3768 | 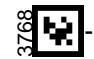   | ↘ | 3769 | 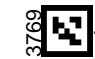   | ↘ | 3772 | 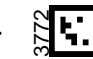   | ↘ | 3773 | 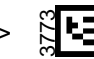   | ↘ | 3792 | 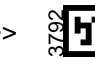   | ↘ | 3793 | 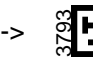   | ↘ | 3796 | 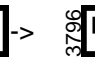   | ↘ | 3797 | 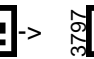   | ↘ |
| 3802 | 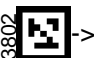   | ↘ | 3803 | 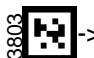   | ↘ | 3806 | 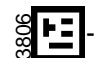   | ↘ | 3807 | 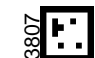   | ↘ | 3808 | 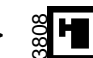   | ↘ | 3823 | 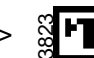   | ↘ | 3824 | 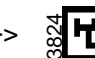   | ↘ | 3825 | 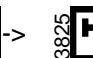   | ↘ | 3829 | 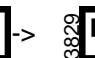   | ↘ | 3834 | 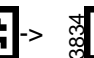   | ↘ |
| 3838 | 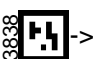 | ↘ | 3839 | 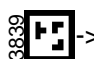 | ↘ | 3856 | 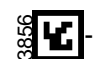 | ↘ | 3861 | 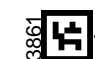 | ↘ | 3866 | 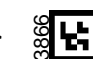 | ↘ | 3871 | 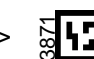 | ↘ | 3888 | 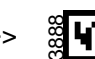 | ↘ | 3889 | 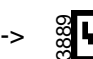 | ↘ | 3892 | 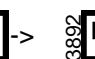 | ↘ | 3893 | 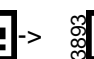 | ↘ |
| 3898 | 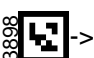 | ↘ | 3899 | 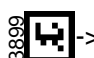 | ↘ | 3902 | 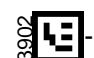 | ↘ | 3903 | 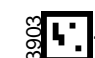 | ↘ | 3922 | 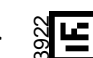 | ↘ | 3923 | 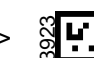 | ↘ | 3926 | 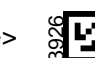 | ↘ | 3927 | 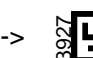 | ↘ | 3928 | 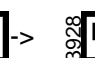 | ↘ | 3929 | 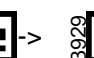 | ↘ |
| 3932 | 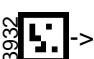 | ↘ | 3933 | 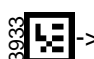 | ↘ | 3954 | 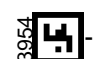 | ↘ | 3955 | 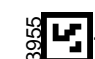 | ↘ | 3958 | 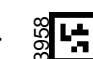 | ↘ | 3961 | 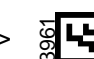 | ↘ | 3963 | 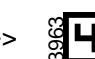 | ↘ | 3964 | 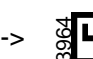 | ↘ | 3965 | 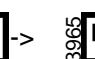 | ↘ | 3984 | 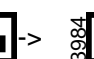 | ↘ |
| 3985 | 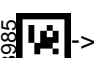 | ↘ | 3988 | 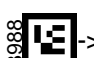 | ↘ | 3989 | 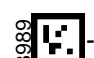 | ↘ | 3994 | 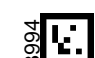 | ↘ | 3995 | 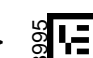 | ↘ | 3998 | 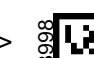 | ↘ | 3999 | 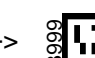 | ↘ | 4016 | 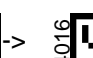 | ↘ | 4020 | 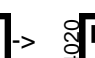 | ↘ | 4021 | 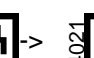 | ↘ |
| 4026 | 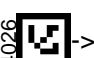 | ↘ | 4027 | 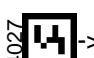 | ↘ | 4030 | 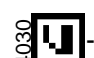 | ↘ | 4031 | 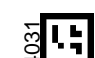 | ↘ | 4051 | 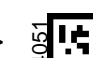 | ↘ | 4054 | 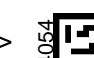 | ↘ | 4055 | 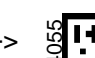 | ↘ | 4056 | 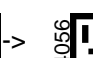 | ↘ | 4057 | 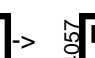 | ↘ | 4060 | 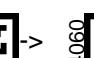 | ↘ |

Supplement: S1 Code Supplement — Functions and dependencies associated with the BEEtag tracking software for Matlab. (ZIP) [file pone.0136487.s001.zip › BEEtag-master/src/900-999keyed.pdf]

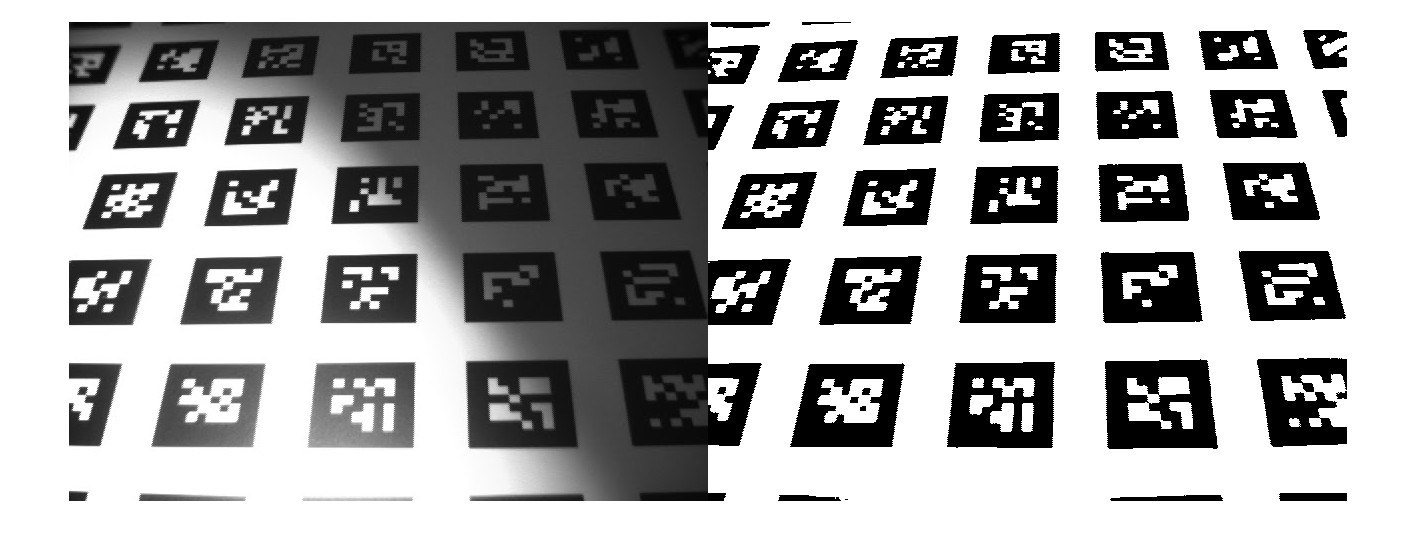

Supplement: S1 Code Supplement — Functions and dependencies associated with the BEEtag tracking software for Matlab. (ZIP) [file pone.0136487.s001.zip › BEEtag-master/src/bradley/bradley/bradley.png]
